# Implementing a nicotine cap to make America Healthy Again

**DOI:** 10.1093/haschl/qxag239

**Published:** 2026-09-03

**Authors:** Dana Mowls Carroll, Dorothy K Hatsukami, Olivia A Wackowski, Francis Joseph McClernon, Eric Donny, Mitch Zeller

**Affiliations:** Masonic Cancer Center, University of Minnesota, Minneapolis, MN 55455, United States; Masonic Cancer Center, University of Minnesota, Minneapolis, MN 55455, United States; Rutgers Institute for Nicotine & Tobacco Studies, Rutgers University, New Brunswick, NJ 08901, United States; Department of Psychiatry and Behavioral Sciences, Duke University School of Medicine, Durham, NC 27710, United States; Department of Translational Neuroscience, Wake Forest University School of Medicine, Winston-Salem, NC 27157, United States; Center for Tobacco Products, Food and Drug Administration, Silver Spring, MD 20993, United States

**Keywords:** Nicotine standard, smoking cessation, tobacco, chronic disease, cancer, tobacco control policy, continuum of risk, relative risks, public health policy, cigarettes, addiction, make America healthy again, MAHA

## Abstract

The US Department of Health and Human Services’ (HHS) “Make America Healthy Again” initiative cannot eradicate the root causes of the nation's chronic disease epidemic without tackling cigarette smoking, which kills over 480 000 Americans and costs $600 billion annually. This commentary reinvigorates a pivotal 2017 FDA framework by urging the current administration to finalize the proposed rule establishing a maximum nicotine cap in all cigarettes and selected combusted tobacco. Evidence demonstrates that capping nicotine at nonaddictive levels (∼95% reduction) rapidly decreases dependence and accelerates smoking cessation. The FDA projects that a nicotine cap will prevent 4.3 million smoking-related deaths by 2100 while saving $1.1 trillion annually in healthcare and productivity costs over 40 years. To maximize these gains, we outline 2 supporting strategies. First, the administration must substantially fund the nation's under-resourced smoking cessation infrastructure to meet the inevitable surge in quitting. Second, provide adults who smoke evidence-based information on the full spectrum of quitting smoking options by communicating the tobacco/nicotine continuum of risk, that is, that combusted products (eg, cigarette) carry the greatest risk of disease, non-combusted products (eg, e-cigarettes) are likely to pose less risk than cigarettes, and medicinal nicotine products (eg, patches) are the safest option.

Key points

- To “Make America Healthy Again” the current US administration must tackle the addictiveness of its most lethal consumer product by implementing a nicotine cap in cigarettes and select other combustible tobacco products.
- Support efforts, which include increasing funding for and access to cessation infrastructure and providing education to adults who smoke on the tobacco/nicotine continuum of risk, are recommended alongside a nicotine cap.

The US Department of Health and Human Services (HHS) embarked on the “Make America Healthy Again” initiative to eradicate the root causes of the American chronic disease epidemic. We believe that the goal of this initiative cannot be achieved without addressing the greatest cause of preventable chronic disease—cigarette smoking. As of 2025, 27 million Americans smoke cigarettes.^1^ Smoking costs over 480 000 American lives each year, untold amounts of morbidity and pain, and a staggering $600 billion annually in healthcare expenses and lost productivity.^2^ Importantly, ending combustible tobacco use directly aligns with the hopes of many Americans who smoke given that each year approximately two-thirds want to quit and over half try, yet <10% succeed.^3^

The blueprint for addressing cigarette smoking already exists within the current administration's past actions. In 2017, during the first Trump Administration, a framework by Scott Gottlieb, the then–US Commissioner of the FDA, and Mitch Zeller, the then–Director of the FDA Center for Tobacco Products (CTP), outlined a pivotal strategy for tobacco regulatory policy.^4^ The underlying principle of that strategy remains more relevant than ever: “reducing the addictiveness of combustible cigarettes while recognizing and clarifying the role that potentially less harmful tobacco products could play in improving public health.” We seek to reinvigorate that strategy by recommending the finalization of the single most important action that the HHS could do to make America healthier: capping the nicotine content in all cigarettes and other selected combusted tobacco at minimally or nonaddictive levels. To *maximize* the success of a nicotine cap, which was recently (in 2025) proposed by the FDA,^5^ we recommend that a nicotine cap be supported with 2 efforts: (1) increased access to smoking cessation supports and (2) education on the tobacco/nicotine continuum of risk.

## Nicotine cap

The most impactful lever for getting Americans off cigarettes and improving their health is implementing a nicotine cap on all cigarettes and other combustible tobacco products. The FDA CTP has the authority to regulate tobacco products for the protection of public health. Under this authority, the FDA can require tobacco product standards that would cap nicotine levels, as long as the level is not reduced to zero. Rigorous and replicated research, summarized previously by the Society for Research on Nicotine and Tobacco,^6^ demonstrates that cigarettes containing ∼95% less nicotine reduce cigarette dependence, lower cigarettes smoked per day, and increase quitting smoking. FDA's own estimates are that by 2100, this single policy could prevent 4.3 million smoking caused deaths and lead to almost 77 million additional years of life.^5^ To put that in perspective, it is the equivalent to preventing every single death in America for over a year, or sparing a population larger than the state of Oklahoma. It is also projected to save $1.1 trillion annually in healthcare and productivity costs for the first 40 years.^5^ Importantly, evidence from multiple studies shows that the majority of people who smoke support this policy,^6,7^ with the highest levels of support occurring when framed as a tool to prevent youth addiction to cigarettes and facilitate cessation.^7^

Implementation of the nicotine cap is within our reach. FDA published a proposed nicotine cap rule in January 2025.^5^ The comment period closed in September 2025, and the rule has not been withdrawn. It awaits a decision by the Administration whether to proceed with a final rule. We urge the HHS through the FDA to finalize the rule.

## Increase access to smoking cessation supports

A nicotine cap in cigarettes would represent the nation's greatest opportunity to quit smoking. This policy will drive a substantial number of people to make quit attempts (∼19.5 million Americans are estimated to quit within 5 years based on the FDA's own modeling^5^), efforts that can be significantly amplified by the strategic provision of cessation supports. We are fortunate that America has an infrastructure of relevant supports that can be leveraged, comprising successful media campaigns (eg, Tips From Former Smokers), a nationwide network of state-run quitlines (1-800-QUIT-NOW), and digital platforms like Smokefree.gov. These resources are increasingly interconnected, which mean people can easily access the resources and support they need, regardless of the first step they take. Additionally, Medicaid serves as the primary vehicle for delivering smoking cessation support to lower-income Americans—the demographic that bears the highest burden of tobacco use. Complementing these systems are health care providers, including local pharmacies, who serve as a key entry point to smoking cessation supports.

The supports above, while proven to work, are currently seriously underfunded and would not meet the demand for them following enacting a nicotine cap. Increasing funding for and access to these cessation resources would help ensure that the benefits of a nicotine cap can be maximal.

## Educate people who smoke on the tobacco/nicotine continuum of risk

In the wake of a nicotine cap, Americans deserve transparent, evidence-based information on the full spectrum of quitting options. The best option is to quit smoking with the aid of FDA-approved smoking cessation medications and existing support resources. However, many Americans will still find it a challenge to quit tobacco entirely, including those who have tried but have not had success with traditional methods like nicotine medicines, and may benefit from trying/switching to other lower-risk nicotine products. Thus, we recommend clearly communicating the continuum of risk of tobacco/nicotine products—that is, help adults who smoke understand that combusted tobacco products (eg, cigarettes) carry the greatest risk of disease, non-combusted products such as e-cigarettes are likely to carry less risk of disease than cigarettes, and medicinal nicotine products(eg, patches) are the safest option. We recommend this communication because there are widespread misperceptions on the health risks of non-combusted tobacco/nicotine products that likely impede people who smoke from completely switching to them and thus quitting smoking. Specifically, many people who smoke believe that non-combusted products are as harmful or even more harmful than cigarettes.^8^ To minimize inadvertently prompting youth and nonusers to initiate use, these communications should be targeted directly to adults who smoke via clinical provider advice, state quitlines, and digital cessation platforms.

Importantly, communication about the continuum of risk is in direct alignment with the FDA CTP's 2023 Strategic Plan and their website on this topic.^9^ This approach is also reflected in a 2024 commentary coauthored by the former FDA CTP Director on considerations for healthcare providers in talking with patients who do not succeed with traditional approaches about switching to tobacco/nicotine products with lower health risks,^10^ and a 2025 FDA press release on the role of less harmful tobacco/nicotine products in maximizing the positive health impacts of a nicotine cap.^5^ In addition to being likely to carry lower risk of disease, some products have evidence for smoking cessation. A 2025 Cochrane Systematic Review found “high certainty” evidence that e-cigarettes are even more effective than nicotine medicines.^11^

Additional considerations are outlined in Appendix A.

## Summary

Implementing a nicotine cap in cigarettes is the most important action that could be taken to address America's chronic disease epidemic, and we recommend moving this policy forward, along with 2 efforts (access to smoking cessation resources and targeted education on the tobacco/nicotine continuum of risk) to support its impact.

## Supplementary Material

qxag239_Supplementary_Data
