# Supplementary material for: Implementing a nicotine cap to make America Healthy Again
Source: Health Aff Sch. 2026 Sep 3;4(9):qxag239. doi: 10.1093/haschl/qxag239 (PMC13602413; doi:10.1093/haschl/qxag239)
Supplement: Supplementary file 1 [file qxag239_supplementary_data.zip › Additional considerations 06.03.2026.docx]

***Appendix A***

***Additional considerations***

There are a few additional points to raise when considering implementing a nicotine cap. First, while concerns regarding a "black market" are often raised, our research indicates that most people who smoke are unlikely to seek out illicit, normal nicotine, cigarettes.(1) Furthermore, risk of an illicit market can be mitigated through provision of the two support strategies: providing robust access to cessation tools and transparent information on lower-risk nicotine alternatives. Second, though the overall smoking prevalence is at a historic low of 10%, now is the strategic moment to act. A nicotine cap prevents the pendulum from swinging back and ensures that we finally eliminate the most lethal consumer product in the American marketplace. We should not settle for a "low" epidemic when we have the tools to end it. Additionally, the overall prevalence of smoking (10%) masks the reality of higher smoking among several U.S. populations. For example, smoking is estimated to be at 15% among rural residing Americans and 18% among those who did not graduate high school.(4) Third, smoking reductions with very low nicotine content cigarettes have been observed regardless of age, race, socio-economic status, mental health conditions, and heaviness of smoking.(5-10) Furthermore, unintended consequences commonly raised,(11) such as smoking cigarettes more intensely, an increase in other substance use (e.g., alcohol or cannabis) or an increase in mental health symptoms, are very rare, limited, or non-existent.(7, 8, 12-14) Forth, the United States has surveillance systems that can monitor the impact of our recommended strategy on public health and cost savings. These include surveys/databases that track tobacco behaviors (e.g., Population Assessment of Tobacco Use and Health, National Youth Tobacco Survey, NielsenIQ point-of-sale) and associated health outcomes (e.g., National Center for Health Statistics). By monitoring these metrics, this Administration can lay claim to the millions of lives and dollars saved in its effort to make America healthy again.

Literature Cited

1.	Hackworth EE, Carroll DM, Donny EC, Denlinger-Apte RL, Hatsukami D. Policy support and potential black-market use in response to a reduced nicotine standard: responses following a 12-week open-label clinical trial. Tobacco control. 2025.

2.	Reynolds RM, Trasher JF, Yang B, Henderson KC, Ashley DL, Hackworth EE, et al. Perceptions of a reduced nicotine policy and predictors of policy support: A nationally representative US survey. Preventive medicine. 2024;184:107952.

3.	Denlinger-Apte RL, Tidey JW, Koopmeiners JS, Hatsukami DK, Smith TT, Pacek LR, et al. Correlates of support for a nicotine-reduction policy in smokers with 6-week exposure to very low nicotine cigarettes. Tobacco control. 2019;28(3):352–5.

4.	American Lung Association. Top 11 Communities Disproportionately Affected by Cigarette Smoking and Tobacco Use 2025 [Available from: <https://www.lung.org/research/sotc/by-the-numbers/top-10-populations-affected>.

5.	Foulds J, Veldheer S, Pachas G, Hrabovsky S, Hameed A, Allen SI, et al. The effects of reduced nicotine content cigarettes on biomarkers of nicotine and toxicant exposure, smoking behavior and psychiatric symptoms in smokers with mood or anxiety disorders: A double-blind randomized trial. PLoS One. 2022;17(11):e0275522.

6.	Cassidy RN, Tidey JW, Jackson KM, Cioe PA, Murphy SE, Krishnan-Sarin S, et al. The impact of reducing nicotine content on adolescent cigarette smoking and nicotine exposure: Results from a randomized controlled trial. Nicotine Tob Res. 2023;25(5):918–27.

7.	Tidey JW, Snell LM, Colby SM, Cassidy RN, Denlinger-Apte RL. Effects of very low nicotine content cigarettes on smoking across vulnerable populations. Prev Med. 2022;165(Pt B):107099.

8.	Higgins ST, Tidey JW, Sigmon SC, Heil SH, Gaalema DE, Lee D, et al. Changes in Cigarette Consumption With Reduced Nicotine Content Cigarettes Among Smokers With Psychiatric Conditions or Socioeconomic Disadvantage: 3 Randomized Clinical Trials. JAMA network open. 2020;3(10):e2019311–e.

9.	Said RJ, Carroll DM, Luo X, Hu J, Cao Q, Tessier KM, et al. Secondary analysis of a randomized clinical trial of very low nicotine cigarettes: Outcomes across social and demographic groups. Preventive medicine. 2025:108362.

10.	Carroll DM, Lindgren BR, Dermody SS, Denlinger-Apte R, Egbert A, Cassidy RN, et al. Impact of nicotine reduction in cigarettes on smoking behavior and exposure: Are there differences by race/ethnicity, educational attainment, or gender? Drug and alcohol dependence. 2021;225:108756.

11.	Denlinger-Apte RL, White CM, Donny EC. What to Expect From a Low-Nicotine Product Standard for Cigarettes: A Response to “Denicotinized” Tobacco. JAMA. 2025;333(5):371–2.

12.	Meier E, Rubin N, Dermody SS, Tessier KM, Hecht SS, Murphy S, et al. Immediate Switching to Reduced Nicotine Cigarettes in a U.S.-Based Sample: The Impact on Cannabis Use and Related Variables at 20 Weeks. Nicotine & tobacco research : official journal of the Society for Research on Nicotine and Tobacco. 2023;25(5):867–74.

13.	Dermody SS, Tessier KM, Meier E, al'Absi M, Denlinger-Apte RL, Drobes DJ, et al. An Evaluation of Potential Unintended Consequences of a Nicotine Product Standard: A Focus on Drinking History and Outcomes. Nicotine & tobacco research : official journal of the Society for Research on Nicotine and Tobacco. 2021;23(7):1168–75.

14.	Dermody SS, Tidey JW, Denlinger RL, Pacek LR, al'Absi M, Drobes DJ, et al. The Impact of Smoking Very Low Nicotine Content Cigarettes on Alcohol Use. Alcohol Clin Exp Res. 2016;40(3):606–15.
